# Watch-and-wait after immunotherapy-containing neoadjuvant treatment in pMMR/MSS locally advanced rectal cancer: a multicenter real-world study

**DOI:** 10.3389/fimmu.2026.1909441

**Published:** 2026-08-10

**Authors:** Zhenkai Luo, Renshen Xiang, Ruihuan Shen, Hang Zhao

**Affiliations:** 1 Department of Endoscopy, Zhejiang Cancer Hospital, Hangzhou Institute of Medicine (HIM), Chinese Academy of Sciences, Hangzhou, Zhejiang, China; 2 Department of Colorectal Oncology, Tianjin’s Clinical Research Center for Cancer, Key Laboratory of Cancer Prevention and Therapy, Tianjin Medical University Cancer Institute and Hospital, National Clinical Research Center for Cancer, Tianjin, China; 3 Department of Translational Medicine and Clinical Research, Sir Run Run Shaw Hospital, Zhejiang University School of Medicine, Hangzhou, Zhejiang, China; 4 Department of Head and Neck Surgery, Fudan University Shanghai Cancer Center, Shanghai, China; 5 Department of Oncology, Shanghai Medical College, Fudan University, Shanghai, China

**Keywords:** clinical complete response, immunotherapy-containing neoadjuvant treatment, locally advanced rectal cancer, pMMR/MSS, watch-and-wait

## Abstract

**Background:**

Watch-and-wait (W&W) is an organ-preserving option for rectal cancer patients who achieve clinical complete response (cCR), but post-cCR outcomes after immunotherapy-containing neoadjuvant treatment in proficient mismatch repair (pMMR)/microsatellite stable (MSS) locally advanced rectal cancer (LARC) remain unclear. We evaluated post-cCR oncological and organ-preservation outcomes in this selected population.

**Methods:**

This multicenter retrospective cohort study included patients with pMMR/MSS LARC who achieved cCR after immunotherapy-containing neoadjuvant treatment at three medical centers and subsequently underwent W&W. Eligible patients had at least 12 months of post-cCR follow-up. Disease progression, survival, and organ-preservation outcomes were evaluated, and survival was estimated using the Kaplan–Meier method.

**Results:**

Twenty-five patients were included, with a median post-cCR follow-up of 26 months (range, 14–40 months). Three patients (12.0%) developed disease progression, including two with local regrowth and one with lung metastasis. Both local regrowths were treated with R0 salvage surgery. The estimated 2-year disease-free survival was 87.4% (95% CI, 65.7%–95.8%). Twenty-three patients (92.0%) avoided radical surgery, and 22 patients (88.0%) maintained disease-free organ preservation.

**Conclusion:**

This multicenter study provides preliminary real-world evidence on W&W after cCR in highly selected patients with pMMR/MSS LARC treated with immunotherapy-containing neoadjuvant regimens, but larger prospective comparative studies with longer follow-up are required before its feasibility and oncological safety can be established.

## Introduction

Locally advanced rectal cancer (LARC) is usually defined as clinical T3–4 or node-positive disease (1, 2). Neoadjuvant chemoradiotherapy followed by total mesorectal excision (TME) remains a standard treatment approach for these patients. Although this strategy improves local control, only approximately 10%–20% of patients achieve a pathological complete response (pCR) after conventional neoadjuvant therapy (3, 4). In addition, radical surgery may be associated with a temporary or permanent stoma, impaired anorectal function, and urinary or sexual dysfunction, all of which may affect quality of life (5–7). Therefore, how to preserve organ function while ensuring tumor control is a critical issue in LARC management.

In recent years, immunotherapy represented by immune checkpoint inhibitors (ICIs) targeting programmed death-1 (PD-1) and programmed death-ligand 1 (PD-L1) has transformed the treatment landscape for colorectal cancer patients with deficient mismatch repair (dMMR)/microsatellite instability-high (MSI-H) due to its remarkable efficacy (8, 9). However, most rectal cancers are proficient mismatch repair (pMMR)/microsatellite stable (MSS) tumors, which generally respond poorly to immunotherapy alone (10, 11). Thus, the application of immunotherapy in pMMR/MSS LARC relies heavily on combination strategies.

Radiotherapy not only directly kills tumor cells but may also enhance the response to immunotherapy by promoting tumor antigen release and remodeling the tumor immune microenvironment (12, 13). Several studies, including UNION, TORCH, and STELLAR II, have evaluated radiotherapy and chemotherapy combined with PD-1 blockade in pMMR/MSS LARC. These studies reported encouraging pCR rates of 39.8%–50.0%, and the TORCH trial reported a clinical complete response (cCR) rate of 43.5% (14–16). These encouraging results have opened the possibility of organ preservation for these patients.

For patients who achieve cCR after neoadjuvant therapy, the watch-and-wait (W&W) strategy has been validated for its safety and efficacy in multiple studies (17–19). In carefully selected patients under intensive surveillance, the W&W strategy may reduce surgical trauma and preserve the rectum while maintaining acceptable oncologic outcomes, although functional benefits require confirmation using standardized patient-reported outcomes. However, existing evidence on W&W following immunotherapy-containing neoadjuvant treatment has predominantly come from patients with dMMR/MSI-H rectal cancer (20). The post-cCR outcomes of pMMR/MSS LARC patients who undergo W&W after immunotherapy-containing neoadjuvant regimens are still not well described.

In this multicenter retrospective real-world cohort study, we evaluated pMMR/MSS LARC patients who achieved cCR after immunotherapy-containing neoadjuvant regimens and were managed with W&W. We focused on post-cCR disease progression, survival status, and organ preservation, with the aim of characterizing post-cCR oncological outcomes and the risks of nonoperative management in this patient population.

## Methods

### Patient selection

This multicenter retrospective real-world cohort study screened patients with pMMR/MSS LARC who received immunotherapy-containing neoadjuvant treatment at three medical centers between June 2019 and June 2025. The present study did not prospectively assign treatment but evaluated post-cCR outcomes among patients who subsequently elected to undergo W&W management. LARC was defined as rectal adenocarcinoma staged as cT3–4 and/or cN+ without distant metastasis based on pretreatment pelvic magnetic resonance imaging (MRI), endoscopy, pathological diagnosis, and systemic imaging. Pretreatment staging was performed using pelvic MRI and chest-abdomen-pelvic computed tomography (CT), with positron emission tomography (PET) performed when clinically indicated. MMR/MSI status was confirmed in all patients by immunohistochemistry (IHC) or next-generation sequencing (NGS). Pretreatment pelvic MRI was reviewed for clinical T/N stage, tumor location, mesorectal fascia (MRF) status, extramural venous invasion (EMVI), and suspicious lateral lymph nodes (LLNs). MRF involvement was defined as tumor-related tissue located within 1 mm of the mesorectal fascia. Tumor deposits were not uniformly reported across centers and were therefore not analyzed separately.

The inclusion criteria were: (1) pathologically confirmed rectal adenocarcinoma; (2) pMMR/MSS status; (3) non-metastatic LARC; (4) receipt of neoadjuvant therapy containing an anti-PD-1/PD-L1 agent; and (5) achievement of cCR followed by a W&W strategy after multidisciplinary team (MDT) assessment. The exclusion criteria were: (1) presence of distant metastasis before treatment; (2) a history of prior immunotherapy or autoimmune disease; (3) follow-up of less than 12 months after cCR; and (4) incomplete clinical data. The data cutoff date was December 2025. Only patients who had completed at least 12 months of post-cCR follow-up by this date were included in the final analysis. The requirement for informed consent was waived due to the retrospective nature of the study, and the study protocol was approved by the ethics committee of each participating center.

### Treatment selection and rationale

The present study was a retrospective multicenter analysis and did not prospectively assign or determine the initial neoadjuvant treatment. According to available records, some patients received immunotherapy-containing neoadjuvant treatment in prospective clinical study settings following corresponding protocols, whereas others received it as an exploratory strategy in routine clinical practice after individualized MDT discussion. Standard options, including upfront surgery when clinically appropriate, conventional chemoradiotherapy, total neoadjuvant therapy, and subsequent TME, were considered and discussed before an immunotherapy-containing regimen was selected. The decision to incorporate immunotherapy was individualized, based on clinical T/N stage, baseline MRI findings, physical condition, and the patient’s willingness to accept a nonstandard exploratory treatment strategy. Immunotherapy combined with chemoradiotherapy (ICRT) was generally considered for patients with low tumors or high-risk features, whereas immunotherapy combined with chemotherapy (ICT) without pelvic radiotherapy was used only in a small number of selected patients with relatively higher tumors, clear MRF, node-negative or low-nodal-burden disease, no suspicious LLNs, and MDT consensus. In some patients without prominent high-risk MRI features, the addition of immunotherapy primarily reflected exploratory institutional practice and individualized decision-making after discussion of its potential benefits, toxicities, nonstandard nature, and uncertain long-term outcomes. Because the original treatment decisions were made independently across centers, no uniform prospectively predefined treatment-selection protocol was applied. The original prospective study identifiers were not reliably retrievable from the available records.

### Neoadjuvant treatment

Neoadjuvant treatment strategies included ICRT and ICT. Radiotherapy included long-course radiotherapy (LCRT; 50 Gy in 25 fractions) or short-course radiotherapy (SCRT; 25 Gy in 5 fractions). In this cohort, SCRT was followed by consolidation systemic therapy rather than immediate surgery. LCRT was delivered with concurrent capecitabine and followed by consolidation systemic therapy when clinically indicated. No patient received induction chemotherapy before radiotherapy. Within the ICRT group, LCRT was generally preferred for patients with T4 disease, N2 disease, MRF involvement, ultra-low tumors, bulky tumors, or other features requiring greater local downstaging, whereas SCRT was selected when a shorter radiotherapy schedule followed by systemic consolidation was considered appropriate.

Systemic chemotherapy consisted of XELOX or FOLFOX. Systemic chemotherapy cycles referred to XELOX or FOLFOX cycles and did not include concurrent capecitabine during LCRT. Anti-PD-1 agents included sintilimab, tislelizumab, pembrolizumab, and camrelizumab, and the anti-PD-L1 agent was envafolimab. Patient-level treatment details, standard chemotherapy dosing schedules, and dose modifications are provided in **Supplementary Table 1**. The number of ICI courses was not prospectively fixed and varied according to treatment strategy, response timing, treatment tolerance, and MDT decision.

### Efficacy and safety assessment

cCR was assessed by the MDT using multimodal evaluation, including digital rectal examination (DRE), pelvic MRI, endoscopy, serum carcinoembryonic antigen (CEA), and systemic imaging. Pelvic MRI and endoscopic assessments were interpreted locally by radiologists and endoscopists at each participating center and reviewed by the institutional MDT; no blinded central review was conducted. All included patients were required to meet the study-defined multimodal cCR criteria described below. The Response Evaluation Criteria in Solid Tumors (RECIST version 1.1) (21) was used mainly for radiological response or progression assessment, whereas cCR was not determined by RECIST alone. cCR required no palpable residual tumor on DRE; no endoscopic residual mass, ulceration, or nodular lesion; no definite viable residual tumor on pelvic MRI, including absence of residual intermediate signal or suspicious diffusion restriction, and no suspicious mesorectal or lateral pelvic lymph nodes; non-elevated or non-rising CEA; and no distant metastasis. Acceptable endoscopic findings included a flat white scar, telangiectasia, or complete mucosal normalization. Selective biopsy was performed only when endoscopic or MRI findings were equivocal or suspicious.

For patients receiving radiotherapy, final cCR assessment was performed after completion of planned neoadjuvant treatment rather than immediately after radiotherapy. For SCRT-based treatment, response assessment was delayed until after consolidation systemic therapy and ICIs. For ICT patients, final response assessment was performed after completion of planned systemic therapy. Treatment-related adverse events (TRAEs) were graded according to the Common Terminology Criteria for Adverse Events, version 5.0.

### Watch-and-wait strategy and follow-up

After MDT-confirmed cCR, W&W was selected through shared decision-making. The date of cCR was defined as the date on which cCR was first confirmed by the MDT. Patients were followed every 3 months during the first 2 years after cCR and every 6 months thereafter for up to 5 years. Follow-up included DRE, CEA testing, pelvic MRI, endoscopy, and systemic imaging. Suspected local regrowth or distant metastasis was reassessed by the MDT for appropriate salvage or systemic treatment. Surveillance compliance was assessed according to expected visits in the institutional W&W schedule; on-schedule visits were defined as visits completed within ±4 weeks during the first 2 years and within ±8 weeks thereafter.

### Outcomes and statistical analysis

The starting point for survival analysis was the date of cCR. The primary endpoint was disease-free survival (DFS), defined as the time from cCR to local regrowth, distant metastasis, death from any cause, or last follow-up. Secondary endpoints included overall survival (OS), organ preservation, and disease-free organ preservation. OS was defined as the time from cCR to death from any cause or last follow-up. Organ preservation was defined as avoidance of radical TME, including patients managed with nonoperative treatment and, when applicable, local excision. Disease-free organ preservation was defined as organ preservation without local regrowth, distant metastasis, or death during follow-up. Categorical variables were presented as frequencies and percentages, while continuous variables were presented as medians and ranges. Survival was estimated using the Kaplan–Meier method, with 95% confidence intervals (CIs) calculated using the Greenwood method with log-log transformation when estimable. Given the small cohort and limited number of events, all Kaplan–Meier analyses were considered descriptive, and no comparative hypothesis testing or multivariable survival modeling was performed. All statistical analyses were performed with R software (version 4.4.2).

## Results

### Patient characteristics

A total of 239 patients with LARC treated with immunotherapy-containing neoadjuvant regimens between June 2019 and June 2025 were screened. cCR was documented in 78 patients (32.6%). Among these patients, 42 underwent immediate surgery and 36 selected nonoperative management with W&W after MDT discussion and shared decision-making. Of the 36 patients managed with W&W, 11 were excluded because of post-cCR follow-up of less than 12 months (n = 9) or incomplete clinical data (n = 2), leaving 25 patients in the final analysis.

The final cohort had a median age of 56 years (range, 27–73), and 16 patients (64.0%) were male. The median body mass index (BMI) was 22.0 kg/m² (range, 18.5–26.7). All patients had pMMR/MSS tumors. Four patients (16.0%) had cN0 disease, 20 (80.0%) had cN1 disease, and 1 (4.0%) had cN2 disease. Tumors were located in the distal rectum in 14 patients (56.0%), middle rectum in 10 patients (40.0%), and proximal rectum in 1 patient (4.0%). Pretreatment MRI showed MRF involvement in 6 patients (24.0%), EMVI positivity in 9 patients (36.0%), and suspicious LLN in 1 patient (4.0%). Baseline characteristics are provided in **Table 1**.

**Table 1:** Patient baseline characteristics (N = 25).

Characteristic	n (%)
Age, years, median (range)	56 (27-73)
Sex	
Male	16 (64.0)
Female	9 (36.0)
BMI, kg/m², median (range)	22.0 (18.5-26.7)
ECOG performance status score	
0	19 (76.0)
1	6 (24.0)
MMR/MSI status	
pMMR/MSS	25 (100.0)
Clinical T stage	
T3	20 (80.0)
T4	5 (20.0)
Clinical N stage	
N0	4 (16.0)
N1	20 (80.0)
N2	1 (4.0)
Distance from anal verge, cm, median (range)	5 (1–12)
Tumor location	
Distal, ≤5 cm	14 (56.0)
Middle, 5–10 cm	10 (40.0)
Proximal, >10 cm	1 (4.0)
Serum level of CEA	
≤ 5 ng/mL	17 (68.0)
> 5 ng/mL	8 (32.0)
MRF status	
Clear	19 (76.0)
Involved	6 (24.0)
EMVI status	
Negative	16 (64.0)
Positive	9 (36.0)
Suspicious LLN	
No	24 (96.0)
Yes	1 (4.0)
Neoadjuvant regimen	
SCRT-ICRT	15 (60.0)
LCRT-ICRT	7 (28.0)
ICT	3 (12.0)
Chemotherapy regimen	
XELOX	17 (68.0)
FOLFOX	8 (32.0)
ICI agent	
Sintilimab	15 (60.0)
Tislelizumab	5 (20.0)
Pembrolizumab	3 (12.0)
Camrelizumab	1 (4.0)
Envafolimab	1 (4.0)

BMI, body mass index; ECOG, Eastern Cooperative Oncology Group; MMR, mismatch repair; MSI, microsatellite instability; pMMR, proficient mismatch repair; MSS, microsatellite stable; CEA, carcinoembryonic antigen; MRF, mesorectal fascia; EMVI, extramural venous invasion; LLN, lateral lymph node; SCRT, short-course radiotherapy; LCRT, long-course radiotherapy; ICRT, immunotherapy combined with chemoradiotherapy; ICT, immunotherapy combined with chemotherapy; ICI, immune checkpoint inhibitor.

### Neoadjuvant treatment response

Patient-level treatment characteristics and post-cCR outcomes are summarized in **Table 2**, with detailed treatment regimens in **Supplementary Table 1**. Of the 25 patients, 22 (88.0%) received ICRT and 3 (12.0%) received ICT. Among ICRT patients, 15 received SCRT and 7 received LCRT. Seventeen patients received XELOX and 8 received FOLFOX. The median number of systemic chemotherapy cycles was 4 (range, 3–6). Treatment modification occurred in 4 patients (16.0%), including dose reduction in 3 and treatment delay in 1; no patient discontinued neoadjuvant treatment because of toxicity.

**Table 2:** Patient-level treatment characteristics and post-cCR outcomes.

Patient	Location	Clinical stage	MRI risk features	Neoadjuvant regimen	ICI agent and courses	Post-cCR follow-up, mo	cCR maintained	Progression type	Salvage treatment	Final status
1	Middle	cT3N1	MRF−; EMVI−; LLN−	SCRT-ICRT; XELOX ×3	Sintilimab ×3	26	Yes	None	NA	NED
2	Distal	cT3N1	MRF−; EMVI−; LLN−	SCRT-ICRT; XELOX ×4	Sintilimab ×4	20	Yes	None	NA	NED
3	Distal	cT4aN1	MRF+; EMVI+; LLN−	LCRT-ICRT; FOLFOX ×6	Tislelizumab ×4	31	Yes	None	NA	NED
4	Distal	cT3N2	MRF+; EMVI+; LLN−	LCRT-ICRT; FOLFOX ×6	Sintilimab ×5	26	No	Lung metastasis at 13 mo	Systemic therapy	AWD
5	Distal	cT3N1	MRF−; EMVI−; LLN−	SCRT-ICRT; XELOX ×4	Sintilimab ×4	28	Yes	None	NA	NED
6	Distal	cT3N1	MRF−; EMVI−; LLN−	SCRT-ICRT; XELOX ×3	Tislelizumab ×3	24	Yes	None	NA	NED
7	Middle	cT3N1	MRF−; EMVI−; LLN−	SCRT-ICRT; XELOX ×3	Sintilimab ×2	35	Yes	None	NA	NED
8	Distal	cT3N1	MRF−; EMVI−; LLN+	LCRT-ICRT; XELOX ×4	Sintilimab ×4	40	No	Local regrowth at 10 mo	Salvage surgery, R0	NED
9	Middle	cT3N1	MRF−; EMVI−; LLN−	ICT; XELOX ×4	Envafolimab ×4	38	Yes	None	NA	NED
10	Middle	cT4aN0	MRF+; EMVI+; LLN−	LCRT-ICRT; FOLFOX ×6	Pembrolizumab ×4	22	Yes	None	NA	NED
11	Distal	cT3N1	MRF−; EMVI−; LLN−	SCRT-ICRT; XELOX ×3	Tislelizumab ×3	30	Yes	None	NA	NED
12	Middle	cT4aN1	MRF+; EMVI+; LLN−	LCRT-ICRT; FOLFOX ×6	Sintilimab ×6	34	Yes	None	NA	NED
13	Middle	cT3N1	MRF−; EMVI+; LLN−	SCRT-ICRT; XELOX ×4	Camrelizumab ×4	27	Yes	None	NA	NED
14	Distal	cT3N1	MRF+; EMVI−; LLN−	LCRT-ICRT; XELOX ×4	Sintilimab ×4	19	Yes	None	NA	NED
15	Distal	cT3N1	MRF−; EMVI−; LLN−	SCRT-ICRT; XELOX ×3	Tislelizumab ×3	15	Yes	None	NA	NED
16	Distal	cT3N1	MRF−; EMVI+; LLN−	SCRT-ICRT; FOLFOX ×4	Pembrolizumab ×4	29	Yes	None	NA	NED
17	Distal	cT3N1	MRF−; EMVI−; LLN−	SCRT-ICRT; XELOX ×4	Sintilimab ×4	25	Yes	None	NA	NED
18	Distal	cT3N1	MRF−; EMVI−; LLN−	SCRT-ICRT; XELOX ×3	Sintilimab ×3	37	Yes	None	NA	NED
19	Middle	cT3N1	MRF−; EMVI−; LLN−	SCRT-ICRT; FOLFOX ×4	Pembrolizumab ×4	15	Yes	None	NA	NED
20	Proximal	cT4aN0	MRF−; EMVI+; LLN−	ICT; FOLFOX ×4	Sintilimab ×4	26	Yes	None	NA	NED
21	Distal	cT3N1	MRF−; EMVI−; LLN−	SCRT-ICRT; XELOX ×4	Sintilimab ×4	23	Yes	None	NA	NED
22	Middle	cT3N1	MRF−; EMVI+; LLN−	SCRT-ICRT; XELOX ×4	Sintilimab ×4	33	Yes	None	NA	NED
23	Middle	cT3N0	MRF−; EMVI−; LLN−	ICT; XELOX ×5	Tislelizumab ×5	14	Yes	None	NA	NED
24	Middle	cT3N1	MRF−; EMVI−; LLN−	SCRT-ICRT; XELOX ×4	Sintilimab ×4	24	Yes	None	NA	NED
25	Distal	cT4bN0	MRF+; EMVI+; LLN−	LCRT-ICRT; FOLFOX ×6	Sintilimab ×5	36	No	Local regrowth at 18 mo	Salvage surgery, R0	NED

cCR, clinical complete response; MRI, magnetic resonance imaging; ICI, immune checkpoint inhibitor; MRF, mesorectal fascia; EMVI, extramural venous invasion; LLN, lateral lymph node; SCRT, short-course radiotherapy; LCRT, long-course radiotherapy; ICRT, immunotherapy combined with chemoradiotherapy; ICT, immunotherapy combined with chemotherapy; NA, not applicable; NED, no evidence of disease; AWD, alive with disease.

ICIs included sintilimab in 15 patients, tislelizumab in 5, pembrolizumab in 3, camrelizumab in 1, and envafolimab in 1. The median time from treatment initiation to final cCR confirmation was 5.0 months (range, 3.2–7.2). All patients underwent DRE, endoscopy, pelvic MRI, CEA testing, and systemic imaging before W&W. Selective biopsy was performed in 5 patients, all negative for carcinoma. Among patients receiving pelvic radiotherapy, the median interval from radiotherapy completion to post-radiotherapy response MRI was 12 weeks (range, 10–13), with no assessment earlier than 10 weeks. Details of cCR assessment are shown in **Supplementary Table 2**. One patient developed grade ≥3 leukopenia during neoadjuvant treatment, which resolved after supportive treatment.

### Follow-up and oncologic outcomes

The median post-cCR follow-up was 26 months (range, 14–40 months), and the median total follow-up from the first dose of ICIs was 32.1 months (range, 18.0–44.4 months). Overall, 152 of 197 expected surveillance visits were completed (77.2%), and 144 were completed within the predefined time window (73.1%). Twenty patients (80.0%) completed at least 80% of scheduled visits. Surveillance details are summarized in **Supplementary Table 3**.

**Figure 1:**
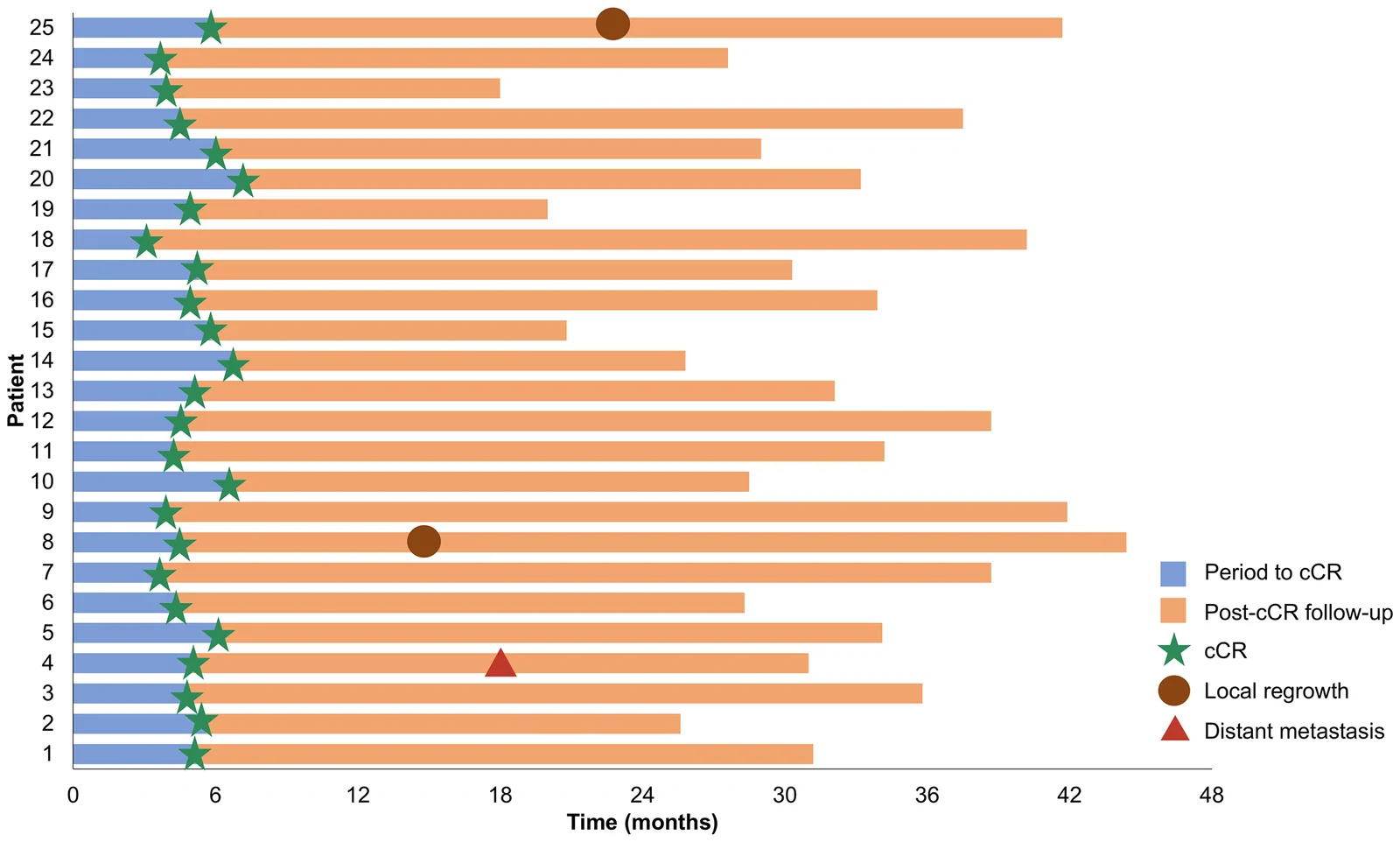
Swimmer plot of patients who received immunotherapy-containing neoadjuvant treatment (N = 25). cCR, clinical complete response.

Disease progression occurred in 3 patients (12.0%). Patient 4 had baseline cT3N2 disease with MRF involvement and EMVI positivity and developed lung metastasis 13 months after cCR, detected by scheduled chest CT; the chest CT 9 months after cCR had been negative. The patient received systemic therapy and was alive with disease at last follow-up. Two patients developed local regrowth. Patient 8 had regrowth 10 months after cCR, detected by pelvic MRI and endoscopy, with MRI suggesting mrT3 disease without suspicious nodal involvement; salvage surgery was performed within 2 weeks, and pathology showed ypT2N0 disease with R0 resection. Patient 25 had regrowth 18 months after cCR, with MRI suggesting mrT3 disease and suspicious nodal involvement; salvage surgery was performed within 3 weeks, and pathology showed ypT3N1 disease with R0 resection. Both patients were alive with no evidence of disease at last follow-up (**Figure 1**).

**Figure 2:**
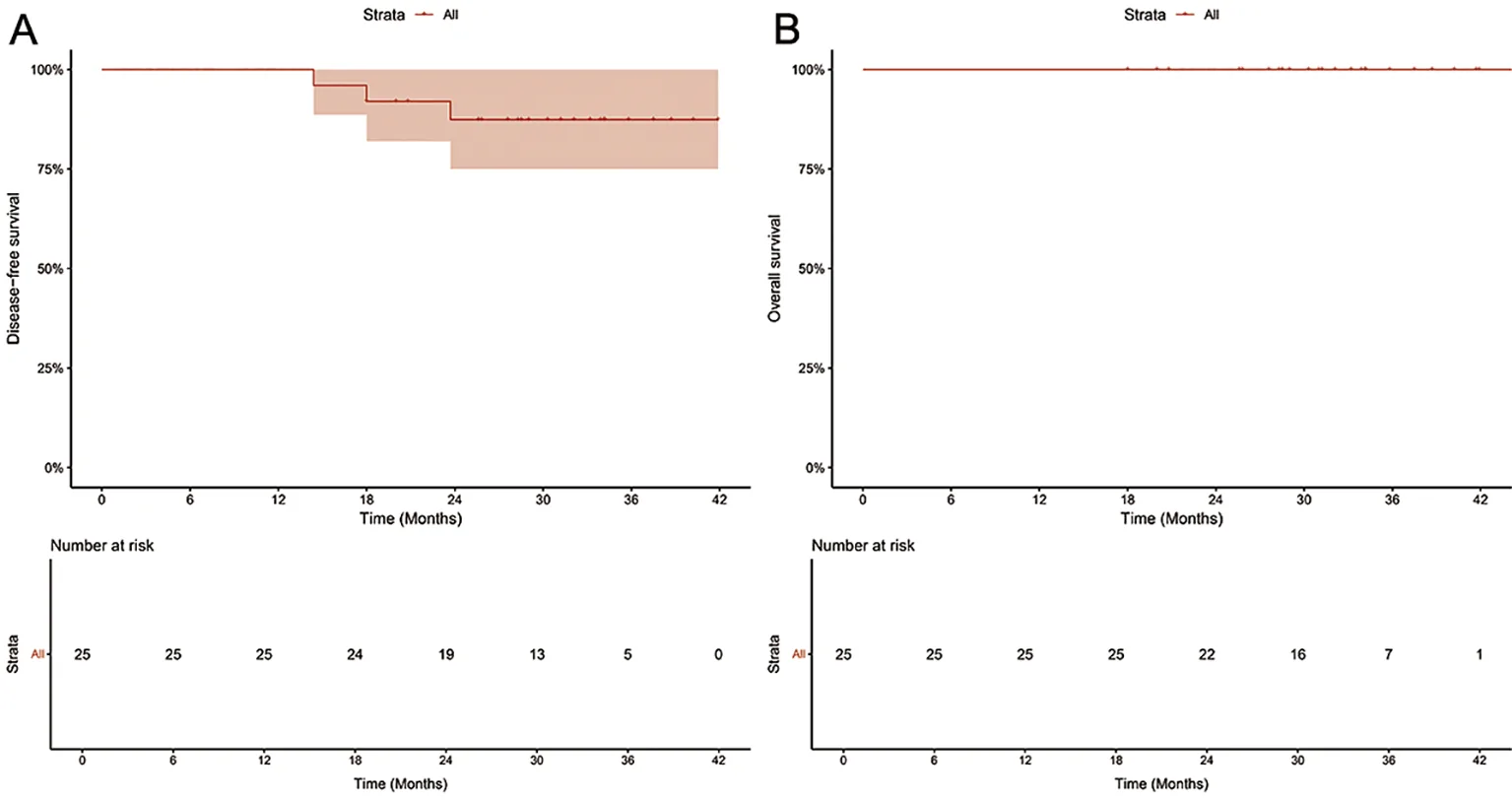
Kaplan–Meier curves for survival outcomes. **(A)** Disease-free survival; **(B)** Overall survival. The estimated 2-year DFS was 87.4% (95% CI, 65.7%–95.8%). No deaths occurred during follow-up; therefore, the 2-year OS was 100.0%, with CI not estimable. Given the limited number of events, these Kaplan–Meier estimates should be interpreted descriptively.

The estimated 2-year DFS was 87.4% (95% CI, 65.7%–95.8%). The 2-year local regrowth-free survival was 91.4% (95% CI, 69.7%–97.8%), and the 2-year distant metastasis-free survival was 96.0% (95% CI, 74.8%–99.4%). No deaths were observed during the current follow-up period; the estimated 2-year OS was 100.0%, and the corresponding CI was not estimable. At the last follow-up, 23 patients (92.0%) had avoided radical surgery and 22 (88.0%) maintained disease-free organ preservation. Descriptive subgroup outcomes, including outcomes by radiotherapy type, N stage, tumor location, MRF status, and ICI agent, are shown in **Supplementary Table 4**, and Kaplan–Meier curves are shown in **Figure 2**.

## Discussion

In this multicenter retrospective study, we evaluated pMMR/MSS LARC patients who achieved cCR after immunotherapy-containing neoadjuvant treatment and were managed with a W&W strategy. During a median post-cCR follow-up of 26 months, disease progression occurred in 3 of 25 patients, including 2 local regrowths and 1 distant metastasis. Both local regrowths were managed with salvage surgery and R0 resection, whereas the patient with distant metastasis remained alive with disease. These findings provide preliminary descriptive evidence regarding W&W in a highly selected post-cCR population, but they do not establish definitive feasibility or oncological safety. The predominance of MRF-negative and cN1 disease in the present cohort should be interpreted in the context of outcome-based inclusion: because only patients who achieved cCR were included, those with less adverse baseline disease may have been more likely to enter the analyzed cohort. This responder enrichment may partly explain the baseline distribution, but it does not justify the initial use of immunotherapy or establish an indication for treatment intensification.

Previous studies of immunotherapy-containing neoadjuvant treatment in pMMR/MSS LARC have mainly focused on tumor regression and pathological response rather than post-cCR nonoperative outcomes. Given the limited activity of ICI monotherapy in pMMR/MSS colorectal cancer, combination strategies using radiotherapy, chemotherapy, and ICIs have been explored to enhance tumor immunogenicity and improve response (10–13, 22, 23). In VOLTAGE-A, chemoradiotherapy followed by nivolumab achieved a pCR rate of 30% in MSS rectal cancer (24). The UNION and TORCH trials also reported encouraging pCR rates with short-course radiotherapy followed by chemotherapy plus anti-PD-1 therapy in pMMR/MSS LARC (14, 15). These responses may reflect radiotherapy-induced immunogenic cell death, tumor-antigen release, and remodeling of the tumor immune microenvironment rather than ICI activity alone (12, 13, 22). Representative historical W&W cohorts and immunotherapy-containing neoadjuvant studies are summarized in **Supplementary Table 5**; however, cross-study comparisons should be interpreted cautiously because of differences in MMR/MSI status, treatment strategy, endpoint definitions, and follow-up duration.

Pathological response and nonoperative management are related but distinct clinical issues. A high postoperative pCR rate indicates substantial tumor regression but does not establish whether patients achieving cCR can safely defer TME. Because eligibility for the present study required both achievement of cCR and selection of W&W, this post-cCR cohort was not designed to estimate the overall cCR rate, compare neoadjuvant regimens, or evaluate the overall efficacy of immunotherapy-containing treatment in an unselected pMMR/MSS LARC population. In this context, DFS, OS, and organ preservation should be interpreted together. Although 23 patients avoided radical TME, 22 maintained disease-free organ preservation. The survival estimates remain descriptive and hypothesis-generating because of the small number of events, wide confidence intervals, and absence of a contemporaneous control group. In particular, the absence of observed deaths during the current follow-up should not be interpreted as evidence of definitive oncological safety.

Disease progression after cCR should be viewed from both local and systemic perspectives. The two local regrowths were detected during surveillance and treated with R0 salvage surgery, supporting the importance of structured follow-up and timely salvage treatment. However, the ypT3N1 finding in one salvage specimen indicates that regrowth after apparent cCR is not always superficial or indolent. This finding also underscores an inherent limitation of current multimodal cCR assessment. DRE, endoscopy, pelvic MRI, CEA, systemic imaging, and selective biopsy cannot completely exclude microscopic residual tumor, deep mural disease, or occult nodal involvement. In particular, superficial endoscopic biopsy, when performed, may not adequately assess deeper mural or nodal disease. cCR should therefore be regarded as a clinical state associated with residual uncertainty rather than as a pathological equivalent of pCR. The single distant metastasis occurred in the only patient with cN2 disease, suggesting that high nodal burden may indicate residual systemic risk despite local cCR. All three progression events occurred in patients with distal tumors, LCRT, and sintilimab exposure, but these observations are descriptive only. Radiotherapy schedule, chemotherapy regimen, ICI agent, and treatment duration were selected jointly and non-randomly according to baseline risk, institutional practice, treatment tolerance, and response timing; therefore, their independent effects on post-cCR outcomes cannot be determined from this cohort. Similarly, the single proximal tumor case represents individualized practice rather than evidence supporting routine W&W for proximal rectal cancer.

Surveillance adherence is critical when interpreting W&W outcomes. In this cohort, 77.2% of expected visits were completed and 73.1% were completed within the predefined time window. Missed or delayed visits may have postponed the detection of local regrowth or distant metastasis, affected the recorded time-to-event, and potentially influenced disease burden at salvage assessment. Therefore, the observed progression rates and timing should be interpreted in the context of incomplete real-world surveillance adherence. The safe implementation of W&W requires reliable multimodal surveillance, patient adherence, experienced MDT assessment, and timely access to salvage treatment, resources that may not be uniformly available across healthcare systems (17, 25). Patient-level cost, reimbursement, and health-economic data were unavailable, precluding formal cost-effectiveness analysis.

W&W following immunotherapy has been better described in dMMR/MSI-H rectal cancer, where PD-1 blockade alone can induce deep and durable responses (8, 20, 26). pMMR/MSS rectal cancer is biologically different and generally responds poorly to ICI monotherapy (10, 27). Therefore, favorable W&W outcomes reported in dMMR/MSI-H rectal cancer should not be directly extrapolated to pMMR/MSS patients. Current guidelines support W&W as a potential management option for carefully selected patients achieving cCR after neoadjuvant therapy (1, 2, 28). However, W&W after immunotherapy-containing neoadjuvant treatment remains investigational in pMMR/MSS LARC and should not be regarded as a standard alternative to surgery.

This study has several limitations. Its retrospective design, non-randomized treatment selection, and inclusion of only patients who achieved cCR and elected W&W resulted in a responder-enriched cohort and introduced substantial selection bias. Immunotherapy-containing neoadjuvant treatment was delivered in heterogeneous clinical contexts without uniform selection criteria, and potential overtreatment in some patients cannot be excluded. The small sample size and limited number of progression events precluded formal subgroup comparisons. Because no contemporaneous control group was included, conclusions regarding comparative efficacy, superiority, or comparative safety cannot be drawn. In patients managed nonoperatively, pathological confirmation was unavailable, and cCR therefore relied on multimodal clinical and radiological assessment; local imaging review may also have introduced interinstitutional variability. Surveillance adherence was incomplete, and standardized patient-reported outcome measures, biomarker data, and health-economic data were not collected. Finally, the median follow-up of 26 months was insufficient to establish long-term oncologic outcomes or the durability of organ preservation; continued follow-up is planned.

## Conclusion

In conclusion, this study provides preliminary multicenter real-world evidence on W&W after cCR in a highly selected pMMR/MSS LARC population treated with immunotherapy-containing neoadjuvant regimens. Local regrowth and distant metastasis occurred despite initial cCR, and the small cohort, treatment heterogeneity, absence of a control group, and limited follow-up preclude definitive conclusions regarding feasibility or oncological safety. This approach should remain investigational pending validation in larger prospective comparative studies with longer follow-up.

## Data Availability

The original contributions presented in the study are included in the article/**Supplementary Material**. Further inquiries can be directed to the corresponding author.
