# Supplementary material for: Watch-and-wait after immunotherapy-containing neoadjuvant treatment in pMMR/MSS locally advanced rectal cancer: a multicenter real-world study
Source: Front Immunol. 2026 Aug 10;17:1909441. doi: 10.3389/fimmu.2026.1909441 (PMC13500611; doi:10.3389/fimmu.2026.1909441)
Supplement: Supplementary file 1 [file Table1.docx]

| **Table S1 Detailed neoadjuvant treatment regimens and treatment modifications** | | | | | | | | | |
| --- | --- | --- | --- | --- | --- | --- | --- | --- | --- |
| Patient | Neoadjuvant regimen | Radiotherapy | Concurrent chemotherapy | Chemotherapy regimen | Chemotherapy timing* | Chemotherapy cycles | Chemotherapy modification | ICI agent | ICI courses |
| 1 | ICRT | SCRT | None | XELOX | Consolidation after SCRT | 3 | None documented | Sintilimab | 3 |
| 2 | ICRT | SCRT | None | XELOX | Consolidation after SCRT | 4 | None documented | Sintilimab | 4 |
| 3 | ICRT | LCRT | CAPE | FOLFOX | Concurrent during LCRT + consolidation after LCRT | 6 | Dose reduction | Tislelizumab | 4 |
| 4 | ICRT | LCRT | CAPE | FOLFOX | Concurrent during LCRT + consolidation after LCRT | 6 | Dose reduction | Sintilimab | 5 |
| 5 | ICRT | SCRT | None | XELOX | Consolidation after SCRT | 4 | None documented | Sintilimab | 4 |
| 6 | ICRT | SCRT | None | XELOX | Consolidation after SCRT | 3 | None documented | Tislelizumab | 3 |
| 7 | ICRT | SCRT | None | XELOX | Consolidation after SCRT | 3 | None documented | Sintilimab | 2 |
| 8 | ICRT | LCRT | CAPE | XELOX | Concurrent during LCRT + consolidation after LCRT | 4 | None documented | Sintilimab | 4 |
| 9 | ICT | NA | None | XELOX | Systemic therapy only | 4 | None documented | Envafolimab | 4 |
| 10 | ICRT | LCRT | CAPE | FOLFOX | Concurrent during LCRT + consolidation after LCRT | 6 | None documented | Pembrolizumab | 4 |
| 11 | ICRT | SCRT | None | XELOX | Consolidation after SCRT | 3 | None documented | Tislelizumab | 3 |
| 12 | ICRT | LCRT | CAPE | FOLFOX | Concurrent during LCRT + consolidation after LCRT | 6 | Treatment delay | Sintilimab | 6 |
| 13 | ICRT | SCRT | None | XELOX | Consolidation after SCRT | 4 | None documented | Camrelizumab | 4 |
| 14 | ICRT | LCRT | CAPE | XELOX | Concurrent during LCRT + consolidation after LCRT | 4 | None documented | Sintilimab | 4 |
| 15 | ICRT | SCRT | None | XELOX | Consolidation after SCRT | 3 | None documented | Tislelizumab | 3 |
| 16 | ICRT | SCRT | None | FOLFOX | Consolidation after SCRT | 4 | None documented | Pembrolizumab | 4 |
| 17 | ICRT | SCRT | None | XELOX | Consolidation after SCRT | 4 | None documented | Sintilimab | 4 |
| 18 | ICRT | SCRT | None | XELOX | Consolidation after SCRT | 3 | None documented | Sintilimab | 3 |
| 19 | ICRT | SCRT | None | FOLFOX | Consolidation after SCRT | 4 | None documented | Pembrolizumab | 4 |
| 20 | ICT | NA | None | FOLFOX | Systemic therapy only | 4 | None documented | Sintilimab | 4 |
| 21 | ICRT | SCRT | None | XELOX | Consolidation after SCRT | 4 | None documented | Sintilimab | 4 |
| 22 | ICRT | SCRT | None | XELOX | Consolidation after SCRT | 4 | None documented | Sintilimab | 4 |
| 23 | ICT | NA | None | XELOX | Systemic therapy only | 5 | None documented | Tislelizumab | 5 |
| 24 | ICRT | SCRT | None | XELOX | Consolidation after SCRT | 4 | None documented | Sintilimab | 4 |
| 25 | ICRT | LCRT | CAPE | FOLFOX | Concurrent during LCRT + consolidation after LCRT | 6 | Dose reduction | Sintilimab | 5 |
| * Standard XELOX consisted of oxaliplatin 130 mg/m² on day 1 plus capecitabine 1000 mg/m² twice daily on days 1–14, every 3 weeks. Standard mFOLFOX6 consisted of oxaliplatin 85 mg/m² and leucovorin 400 mg/m² administered intravenously on day 1, followed by fluorouracil 400 mg/m² as an intravenous bolus and 2,400 mg/m² as a continuous intravenous infusion over 46 hours, repeated every 2 weeks. LCRT was delivered with concurrent capecitabine. ICRT, immunotherapy combined with chemoradiotherapy; ICT, immunotherapy combined with chemotherapy; SCRT, short-course radiotherapy; LCRT, long-course radiotherapy; NA, not applicable; ICI, immune checkpoint inhibitor. | | | | | | | | | |
