# Supplementary material for: Watch-and-wait after immunotherapy-containing neoadjuvant treatment in pMMR/MSS locally advanced rectal cancer: a multicenter real-world study
Source: Front Immunol. 2026 Aug 10;17:1909441. doi: 10.3389/fimmu.2026.1909441 (PMC13500611; doi:10.3389/fimmu.2026.1909441)
Supplement: Supplementary file 2 [file Table2.docx]

| **Table S2 Timing and modalities of cCR assessment before watch-and-wait** | | | | | | | | | | | |
| --- | --- | --- | --- | --- | --- | --- | --- | --- | --- | --- | --- |
| Patient | Treatment strategy | Radiotherapy type | Time to cCR, mo | Post-RT MRI interval, weeks | DRE | Endoscopy | Pelvic MRI | CEA | Systemic imaging | Biopsy performed | Biopsy result |
| 1 | ICRT | SCRT | 5.2 | 10 | Yes | Yes | Yes | Yes | Yes | No | NA |
| 2 | ICRT | SCRT | 5.6 | 12 | Yes | Yes | Yes | Yes | Yes | No | NA |
| 3 | ICRT | LCRT | 4.8 | 12 | Yes | Yes | Yes | Yes | Yes | No | NA |
| 4 | ICRT | LCRT | 5 | 11 | Yes | Yes | Yes | Yes | Yes | No | NA |
| 5 | ICRT | SCRT | 6.1 | 13 | Yes | Yes | Yes | Yes | Yes | No | NA |
| 6 | ICRT | SCRT | 4.3 | 10 | Yes | Yes | Yes | Yes | Yes | No | NA |
| 7 | ICRT | SCRT | 3.7 | 10 | Yes | Yes | Yes | Yes | Yes | No | NA |
| 8 | ICRT | LCRT | 4.4 | 10 | Yes | Yes | Yes | Yes | Yes | Yes | Negative |
| 9 | ICT | No RT | 3.9 | NA | Yes | Yes | Yes | Yes | Yes | No | NA |
| 10 | ICRT | LCRT | 6.5 | 12 | Yes | Yes | Yes | Yes | Yes | Yes | Negative |
| 11 | ICRT | SCRT | 4.2 | 11 | Yes | Yes | Yes | Yes | Yes | No | NA |
| 12 | ICRT | LCRT | 4.7 | 12 | Yes | Yes | Yes | Yes | Yes | Yes | Negative |
| 13 | ICRT | SCRT | 5.1 | 13 | Yes | Yes | Yes | Yes | Yes | No | NA |
| 14 | ICRT | LCRT | 6.8 | 11 | Yes | Yes | Yes | Yes | Yes | Yes | Negative |
| 15 | ICRT | SCRT | 5.8 | 10 | Yes | Yes | Yes | Yes | Yes | No | NA |
| 16 | ICRT | SCRT | 4.9 | 13 | Yes | Yes | Yes | Yes | Yes | No | NA |
| 17 | ICRT | SCRT | 5.3 | 13 | Yes | Yes | Yes | Yes | Yes | No | NA |
| 18 | ICRT | SCRT | 3.2 | 11 | Yes | Yes | Yes | Yes | Yes | No | NA |
| 19 | ICRT | SCRT | 5 | 13 | Yes | Yes | Yes | Yes | Yes | No | NA |
| 20 | ICT | No RT | 7.2 | NA | Yes | Yes | Yes | Yes | Yes | No | NA |
| 21 | ICRT | SCRT | 6 | 12 | Yes | Yes | Yes | Yes | Yes | No | NA |
| 22 | ICRT | SCRT | 4.5 | 13 | Yes | Yes | Yes | Yes | Yes | No | NA |
| 23 | ICT | No RT | 4 | NA | Yes | Yes | Yes | Yes | Yes | No | NA |
| 24 | ICRT | SCRT | 3.6 | 13 | Yes | Yes | Yes | Yes | Yes | No | NA |
| 25 | ICRT | LCRT | 5.7 | 12 | Yes | Yes | Yes | Yes | Yes | Yes | Negative |
| cCR, clinical complete response; RT, radiotherapy; MRI, magnetic resonance imaging; DRE, digital rectal examination; CEA, carcinoembryonic antigen; ICRT, immunotherapy combined with chemoradiotherapy; ICT, immunotherapy combined with chemotherapy; SCRT, short-course radiotherapy; LCRT, long-course radiotherapy; NA, not applicable. | | | | | | | | | | | |
