# Supplementary material for: Watch-and-wait after immunotherapy-containing neoadjuvant treatment in pMMR/MSS locally advanced rectal cancer: a multicenter real-world study
Source: Front Immunol. 2026 Aug 10;17:1909441. doi: 10.3389/fimmu.2026.1909441 (PMC13500611; doi:10.3389/fimmu.2026.1909441)
Supplement: Supplementary file 3 [file Table3.docx]

| **Table S3 Surveillance compliance and examination frequency during watch-and-wait follow-up** | |
| --- | --- |
| Variable | Overall result |
| Post-cCR follow-up, mo, median (range) | 26 (14–40) |
| Expected surveillance visits, n | 197 |
| Completed surveillance visits, n (%) | 152/197 (77.2%) |
| On-schedule surveillance visits, n (%) | 144/197 (73.1%) |
| Patients completing ≥80% of scheduled visits, n (%) | 20 (80.0%) |
| Completed surveillance visits per patient, median (range) | 5 (3–7) |
| DRE per patient, median (range) | 5 (3–7) |
| Pelvic MRI per patient, median (range) | 5 (3–7) |
| Endoscopy per patient, median (range) | 5 (3–7) |
| CEA testing per patient, median (range) | 5 (3–9) |
| Systemic imaging per patient, median (range) | 4 (3–6) |
| cCR, clinical complete response; DRE, digital rectal examination; MRI, magnetic resonance imaging; CEA, carcinoembryonic antigen. | |
