# Supplementary material for: Watch-and-wait after immunotherapy-containing neoadjuvant treatment in pMMR/MSS locally advanced rectal cancer: a multicenter real-world study
Source: Front Immunol. 2026 Aug 10;17:1909441. doi: 10.3389/fimmu.2026.1909441 (PMC13500611; doi:10.3389/fimmu.2026.1909441)
Supplement: Supplementary file 4 [file Table4.docx]

| **Table S4 post-cCR outcomes according to baseline and treatment factors** | | | | | | | | |
| --- | --- | --- | --- | --- | --- | --- | --- | --- |
| Subgroup | Patients, n | Local regrowth, n (%) | Distant metastasis, n (%) | Progression, n (%) | Salvage surgery, n (%) | NED, n (%) | AWD, n (%) | Death, n (%) |
| Overall | 25 | 2 (8.0) | 1 (4.0) | 3 (12.0) | 2 (8.0) | 24 (96.0) | 1 (4.0) | 0 |
| Tumor location |  |  |  |  |  |  |  |  |
| Distal | 14 | 2 (14.3) | 1 (7.1) | 3 (21.4) | 2 (14.3) | 13 (92.9) | 1 (7.1) | 0 |
| Middle | 10 | 0 | 0 | 0 | 0 | 10 (100.0) | 0 | 0 |
| Proximal | 1 | 0 | 0 | 0 | 0 | 1 (100.0) | 0 | 0 |
| Clinical T stage |  |  |  |  |  |  |  |  |
| T3 | 20 | 1 (5.0) | 1 (5.0) | 2 (10.0) | 1 (5.0) | 19 (95.0) | 1 (5.0) | 0 |
| T4 | 5 | 1 (20.0) | 0 | 1 (20.0) | 1 (20.0) | 5 (100.0) | 0 | 0 |
| Clinical N stage |  |  |  |  |  |  |  |  |
| N0 | 4 | 1 (25.0) | 0 | 1 (25.0) | 1 (25.0) | 4 (100.0) | 0 | 0 |
| N1 | 20 | 1 (5.0) | 0 | 1 (5.0) | 1 (5.0) | 20 (100.0) | 0 | 0 |
| N2 | 1 | 0 | 1 (100.0) | 1 (100.0) | 0 | 0 | 1 (100.0) | 0 |
| MRF status |  |  |  |  |  |  |  |  |
| Clear | 19 | 1 (5.3) | 0 | 1 (5.3) | 1 (5.3) | 19 (100.0) | 0 | 0 |
| Involved | 6 | 1 (16.7) | 1 (16.7) | 2 (33.3) | 1 (16.7) | 5 (83.3) | 1 (16.7) | 0 |
| EMVI status |  |  |  |  |  |  |  |  |
| Negative | 16 | 1 (6.2) | 0 | 1 (6.2) | 1 (6.2) | 16 (100.0) | 0 | 0 |
| Positive | 9 | 1 (11.1) | 1 (11.1) | 2 (22.2) | 1 (11.1) | 8 (88.9) | 1 (11.1) | 0 |
| Suspicious LLN |  |  |  |  |  |  |  |  |
| No | 24 | 1 (4.2) | 1 (4.2) | 2 (8.3) | 1 (4.2) | 23 (95.8) | 1 (4.2) | 0 |
| Yes | 1 | 1 (100.0) | 0 | 1 (100.0) | 1 (100.0) | 1 (100.0) | 0 | 0 |
| Neoadjuvant regimen |  |  |  |  |  |  |  |  |
| SCRT-ICRT | 15 | 0 | 0 | 0 | 0 | 15 (100.0) | 0 | 0 |
| LCRT-ICRT | 7 | 2 (28.6) | 1 (14.3) | 3 (42.9) | 2 (28.6) | 6 (85.7) | 1 (14.3) | 0 |
| ICT | 3 | 0 | 0 | 0 | 0 | 3 (100.0) | 0 | 0 |
| Radiotherapy type |  |  |  |  |  |  |  |  |
| SCRT | 15 | 0 | 0 | 0 | 0 | 15 (100.0) | 0 | 0 |
| LCRT | 7 | 2 (28.6) | 1 (14.3) | 3 (42.9) | 2 (28.6) | 6 (85.7) | 1 (14.3) | 0 |
| NA | 3 | 0 | 0 | 0 | 0 | 3 (100.0) | 0 | 0 |
| Chemotherapy regimen |  |  |  |  |  |  |  |  |
| XELOX | 17 | 1 (5.9) | 0 | 1 (5.9) | 1 (5.9) | 17 (100.0) | 0 | 0 |
| FOLFOX | 8 | 1 (12.5) | 1 (12.5) | 2 (25.0) | 1 (12.5) | 7 (87.5) | 1 (12.5) | 0 |
| ICI agent |  |  |  |  |  |  |  |  |
| Sintilimab | 15 | 2 (13.3) | 1 (6.7) | 3 (20.0) | 2 (13.3) | 14 (93.3) | 1 (6.7) | 0 |
| Tislelizumab | 5 | 0 | 0 | 0 | 0 | 5 (100.0) | 0 | 0 |
| Pembrolizumab | 3 | 0 | 0 | 0 | 0 | 3 (100.0) | 0 | 0 |
| Camrelizumab | 1 | 0 | 0 | 0 | 0 | 1 (100.0) | 0 | 0 |
| Envafolimab | 1 | 0 | 0 | 0 | 0 | 1 (100.0) | 0 | 0 |
| ICI class |  |  |  |  |  |  |  |  |
| PD-1 | 24 | 2 (8.3) | 1 (4.2) | 3 (12.5) | 2 (8.3) | 23 (95.8) | 1 (4.2) | 0 |
| PD-L1 | 1 | 0 | 0 | 0 | 0 | 1 (100.0) | 0 | 0 |
| cCR, clinical complete response; AWD, alive with disease; NED, no evidence of disease; MRF, mesorectal fascia; EMVI, extramural venous invasion; LLN, lateral lymph node; SCRT, short-course radiotherapy; LCRT, long-course radiotherapy; ICRT, immunotherapy combined with chemoradiotherapy; ICT, immunotherapy combined with chemotherapy; NA, not applicable; ICI, immune checkpoint inhibitor. | | | | | | | | |
