# Supplementary material for: Watch-and-wait after immunotherapy-containing neoadjuvant treatment in pMMR/MSS locally advanced rectal cancer: a multicenter real-world study
Source: Front Immunol. 2026 Aug 10;17:1909441. doi: 10.3389/fimmu.2026.1909441 (PMC13500611; doi:10.3389/fimmu.2026.1909441)
Supplement: Supplementary file 5 [file Table5.docx]

| **Table S5 Historical comparison of oncologic outcomes in cCR/W&W and immunotherapy-containing rectal cancer cohorts** | | | | | | | | |
| --- | --- | --- | --- | --- | --- | --- | --- | --- |
| Study | Design | Population | n | MMR Status | Neoadjuvant Regimen | pCR/cCR Rate | Survival Outcomes | Key limitation for comparison |
| Present Study | Retrospective, multicenter | pMMR/MSS LARC patients achieving cCR and choosing W&W | 25 | pMMR/MSS | Immunotherapy-containing neoadjuvant treatment: ICRT or ICT | cCR: 100% (by selection) | 2-year DFS: 87.4%; OS: 100% | Small selected post-cCR W&W cohort; no surgical control; treatment heterogeneity |
| UNION (2024) | Phase III RCT | LARC | 231 | Predominantly pMMR/MSS | SCRT followed by CAPOX + camrelizumab vs LCRT followed by CAPOX | pCR 39.8% vs 15.3% | 3-year EFS and OS were immature at early report | Relevant pMMR/MSS immunotherapy reference, but early report focused on pCR rather than post-cCR W&W outcomes |
| TORCH (2024) | Phase II RCT | pMMR/MSS LARC | 121 | pMMR/MSS | Immunotherapy-based TNT with PD-1 inhibitor | CR 56.5% vs 54.2%; cCR 43.5% vs 35.6 | Mature 2-year DFS/OS not reported | Highly relevant pMMR/MSS immunotherapy-based TNT study, but not a pure post-cCR W&W cohort |
| VOLTAGE-A (2023) | Phase II, single-arm | LARC treated with CRT followed by nivolumab and surgery | 42 | MSS and MSI-H reported separately | CRT followed by nivolumab | pCR: MSS 30%, MSI-H 60% | 3-year RFS/OS: 79.5%/97.4% in MSS; 100%/100% in MSI-H | Immunotherapy-containing regimen with MMR/MSI stratification, but surgery-based rather than W&W |
| IWWD (2021) | International registry | Rectal cancer patients with cCR after neoadjuvant therapy | 880 | Mixed | Variable (nCRT/TNT) | cCR: 100% (by selection) | 5-year OS 85%; 5-year DSS 94% | Large W&W reference cohort, but mostly non-immunotherapy era and MMR/MSI not stratified |
| cCR, clinical complete response; W&W, watch-and-wait; LARC, locally advanced rectal cancer; pMMR, proficient mismatch repair; MSS, microsatellite stable; MSI-H, microsatellite instability-high; ICRT, immunotherapy combined with chemoradiotherapy; ICT, immunotherapy combined with chemotherapy; SCRT, short-course radiotherapy; LCRT, long-course radiotherapy; CAPOX, capecitabine plus oxaliplatin; CRT, chemoradiotherapy; TNT, total neoadjuvant therapy; nCRT, neoadjuvant chemoradiotherapy; DFS, disease-free survival; OS, overall survival; EFS, event-free survival; RFS, recurrence-free survival; DSS, disease-specific survival; RCT, randomized controlled trial; pCR, pathological complete response. | | | | | | | | |
